# Endoscopic Treatment of a Gastrocutaneous Fistula Using the Over-The-Scope-Clip System: A Case Report

**DOI:** 10.1155/2011/384143

**Published:** 2011-05-29

**Authors:** Georgios Kouklakis, Petros Zezos, Nikolaos Liratzopoulos, Anthia Gatopoulou, Anastasia Oikonomou, Michail Pitiakoudis, Eleni Efremidou, Constantinos Simopoulos

**Affiliations:** ^1^Endoscopy Unit, University General Hospital of Alexandroupolis, Democritus University of Thrace, Dragana 68100, Alexandroupolis, Greece; ^2^1st Department of Surgery, University General Hospital of Alexandroupolis, Democritus University of Thrace, Dragana 68100, Alexandroupolis, Greece; ^3^Radiology Department, University General Hospital of Alexandroupolis, Democritus University of Thrace, Dragana 68100, Alexandroupolis, Greece; ^4^2nd Department of Surgery, University General Hospital of Alexandroupolis, Democritus University of Thrace, Dragana 68100, Alexandroupolis, Greece

## Abstract

The over-the-scope-clip (OTSC; Ovesco Endoscopy GmbH, Tuebingen, Germany) system is a newly designed method for the mechanical compression of large areas in the gastrointestinal tract. So far, indications for OTSC application are hemostasis of primary or postinterventional bleeding, closure of iatrogenic full-thickness or covered perforations. Recently closure of gastrointestinal tract fistulas using this device has been described. A 44-year-old man developed a gastrocutaneous fistula after surgical treatment for a perforated gastric ulcer. We describe the successful endoscopic closure of the fistula using the OTSC system. The patient's clinical followup was uneventful. Fistula closure was successfully implemented as it was documented by imaging and endoscopic examinations performed on the 2nd day and 6th week after the application of the clip. Endoscopic application of the OTSC device was safe and effective for the treatment of a gastrocutaneous fistula.

## 1. Introduction

The over-the-scope-clip (OTSC) system (Ovesco Endoscopy GmbH, Tuebingen, Germany) is a newly designed method for the mechanical compression of large areas in the gastrointestinal tract. So far, indications for OTSC application are hemostasis primary or postinterventional bleeding and closure of iatrogenic full-thickness or covered perforations during endoscopic mucosal resection or after natural orifice transluminal endoscopic surgery procedures (NOTES) [1].

We report the successful treatment of a gastrocutaneous fistula with closure of the gastric orifice using the OTSC system without postprocedure complications.

## 2. Case Presentation

**Figure 1:**
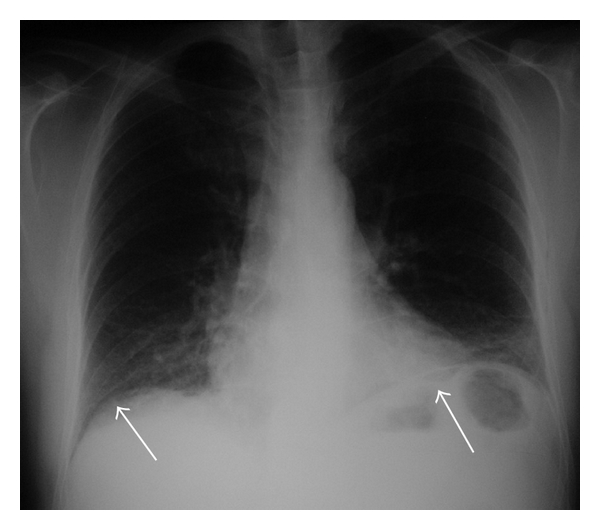
Upright posteroanterior chest radiograph: there is free subdiaphragmatic air bilaterally that is more clearly noted on the left side (white arrows).

A 44-year-old man, who had been having a history of recurrent abdominal pain, attended the emergency services with peritonitis. Upright chest X-ray demonstrated free subdiaphragmatic air bilaterally (Figure 1). An emergent laparotomy was performed and a perforated pyloric ulcer was found which was treated with simple surgical sutures and omental patches. 

**Figure 2:**
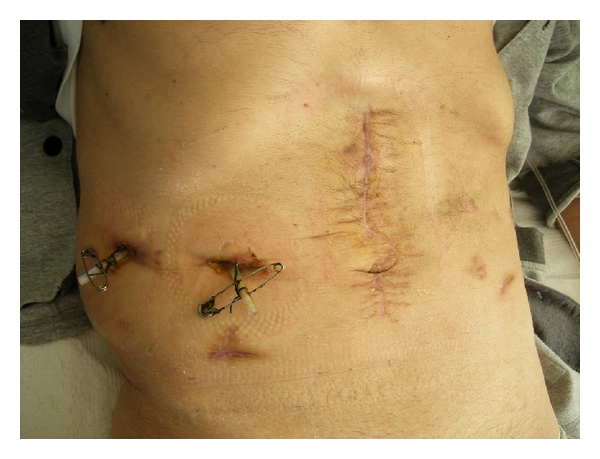
The cutaneous orifice of the fistula discharging gastric juice.

**Figure 3:**
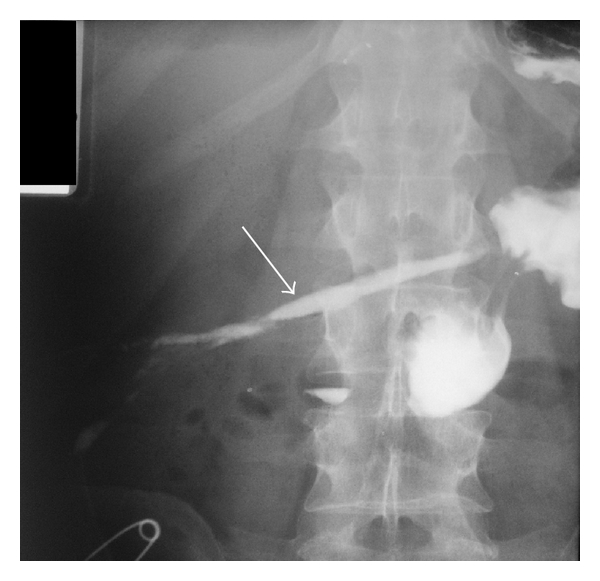
Upper GI study with gastrografin showing a gastrocutaneous fistula (arrow).

A few days later the patient displayed a septic course with persistent fever and leukocytosis. An abdominal CT scanning revealed an abscess in the left subphrenic space and in the space of Douglas. A new surgical intervention was undertaken and peritoneal drainage was reestablished in the areas with the abscesses. The immediate postoperative period was uneventful with the exception of a persistent draining of gastric contents by the abdominal drains (Figure 2). A high-output gastrocutaneous fistula from the lesser curvature was documented on upper gastrointestinal (GI) series with gastrografin (Figure 3), and an upper GI endoscopy revealed the presence of an orifice at the anterior gastric wall located towards the lesser curvature, between the gastric body and the antrum. Biopsies from the gastric orifice revealed nonspecific inflammatory infiltration without evidence of malignancy. 

**Figure 4:**
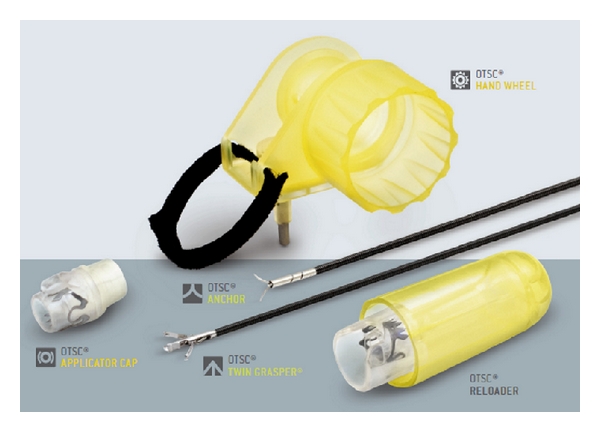
The OTSC system.

**Figure 5:**
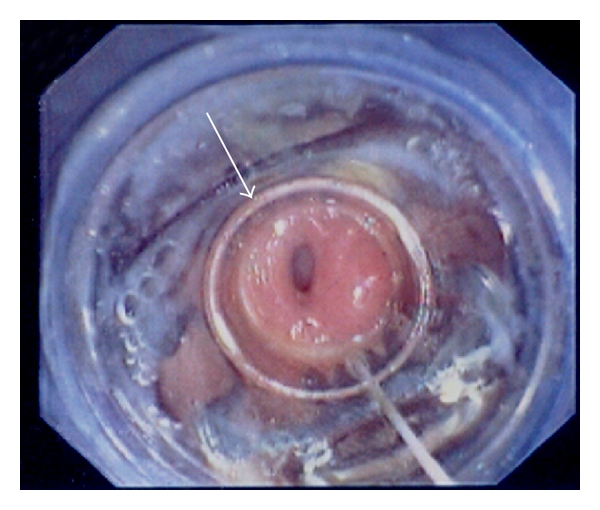
The gastric orifice of the gastrocutaneous fistula (arrow) during endoscopic application of the OTSC device.

**Figure 6:**
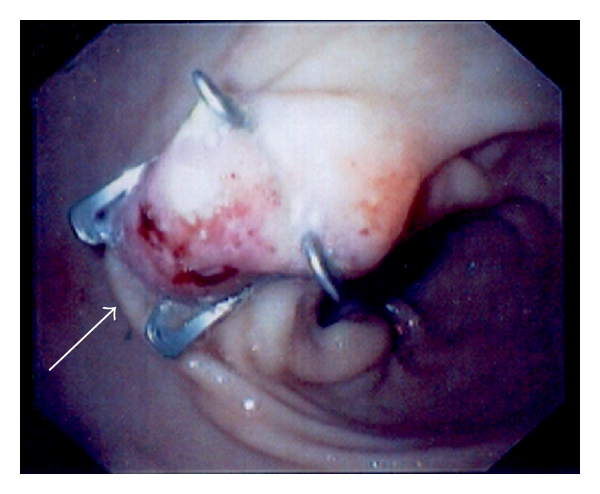
Immediate postinterventional endoscopic view. The tissue at the gastric orifice was captured with OTSC, and the fistula orifice was successfully sealed (arrow).

The gastrocutaneous fistula was persistent after 6 weeks of conservative management with external drainage, gastric decompression with nasogastric catheter, parenteral nutrition, antisecretory drugs, and systemic antibiotics, including those indicated for Helicobacter pylori eradication. Finally, the endoscopic treatment of the fistula was decided using the “traumatic type” OTSC system for the closure of the gastric orifice (Figure 4), and a written informed consent from the patient and the approval by the hospital's medical scientific committee were obtained for this purpose. Upper GI endoscopy was performed, and the endoscope with the mounted and loaded clipping device was inserted into the stomach. The gastric orifice of the fistula was recognized (Figure 5), the tip of endoscope was adapted to the lesion, and additional tissue retraction was obtained by means of the OTSC twin-grasper. The tissue was suctioned into the applicator cap, and the “traumatic” clip was fired once the target tissue was inside the cap. Finally, the orifice and the surrounding tissue that were successfully captured within clip jaws and teeth (Figure 6).

**Figure 7:**
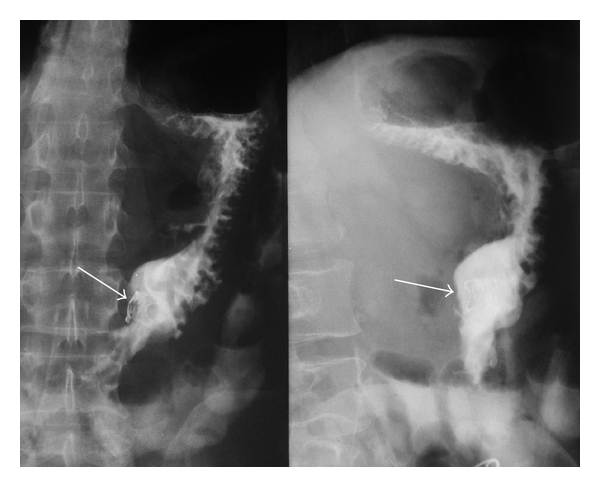
Upper GI study with gastrografin 48 hours after procedure, showing the gastric OTSC in place (arrows) without leaks of contrast media.

**Figure 8:**
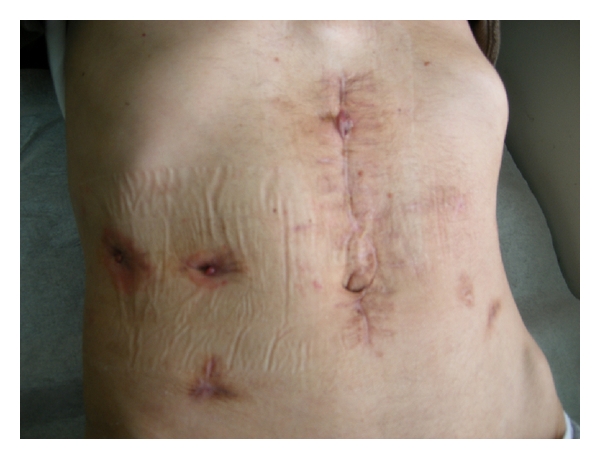
Postinterventional followup after 6 weeks. The cutaneous orifice was completely healed.

**Figure 9:**
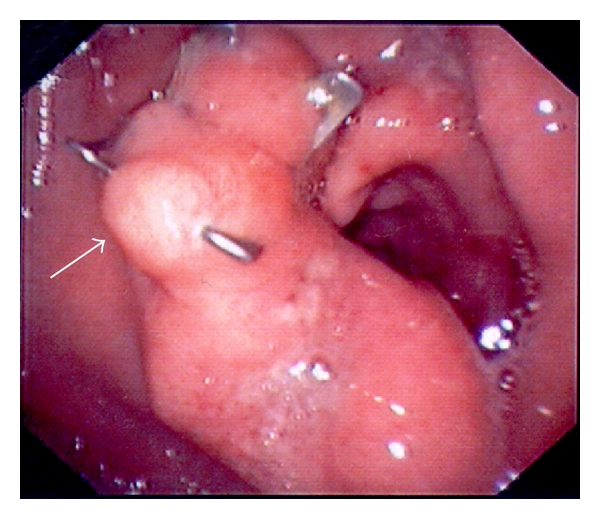
Postinterventional followup after 6 weeks. Endoscopic view of the OTSC in situ with gastric fistula orifice closure (arrow).

**Figure 10:**
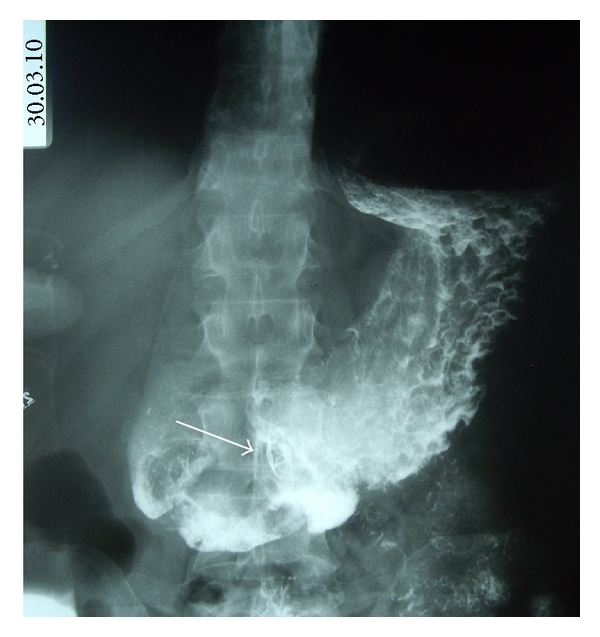
Postinterventional followup after 6 weeks. Gastrografin upper GI study showing the gastric OTSC in place (arrow) without leaks of contrast media.

Upper GI studies with gastrografin (Figure 7) and endoscopy 48 hours after the OTSC placement confirmed the fistula's closure. The postprocedure period was uneventful, the patient was switched to oral diet, and the cutaneous orifice of the fistula was completely healed 2 weeks later (Figure 8). Six weeks after the application of OTSC, upper GI studies with gastrografin swallow (Figure 9) and endoscopy (Figure 10) ruled out any evidence of fistula's persistence or relapse, while the OTSC device was still in place. 

## 3. Case Discussion

Gastrocutaneous fistulas are uncommon complications accounting for 0.5%–3.9% of gastric operations [2]. Gastrocutaneous fistulas after bariatric surgery constitute 25–50% of gastrointestinal ones [3]. Additionally, the persistence of the iatrogenic gastrocutaneous fistula after removal of the percutaneous endoscopic gastrostomy (PEG) tube is a well-known complication [4]. Gastrocutaneous fistulas, which rarely close spontaneously, are related with sepsis, and their management is often difficult and ineffective with high mortality rate [5]. Additionally, persistent leakage from this fistula can lead to severe irritation of the adjacent skin and the soft tissue of the abdominal wall resulting in the development of cellulitis [6].

 A firm etiology for the development of the gastrocutaneous fistula in our patient is difficult to establish. It may be related with a direct or indirect iatrogenic injury or with the inflammatory reaction due to abscess formation adjacent to the stomach. On the other hand, the spontaneous development of a fistula due to a gastric stress ulcer cannot be excluded. 

The optimal approach to the management of gastrocutaneous fistulas remains controversial. Conservative management including adequate drainage, control of infection and sepsis, gastric decompression, gastric acid inhibition, bowel rest, and parenteral nutrition is preferred by most surgeons before surgical intervention [3]. 

Recently, several endoscopic methods have been described for the treatment of gastrocutaneous fistulas including argon plasma coagulation, electrical and chemical cauterization of the fistula, fibrin glue sealant, endoscopic clipping or suturing, or simple suturing [3, 6–8]. The treatment of gastrocutaneous fistulas with biological glue has been reported on different occasions [3, 9, 10]. Papavramidis et al. demonstrated the endoscopic application of a fibrin sealant under direct vision via a double lumen catheter passed through a forward viewing endoscope, safely and effectively [3]. Alberti-Flor described a successful closure of a gastrocutaneous fistula by using percutaneous endoscopic suturing in two patients [11]. Eskaros et al. used a similar, modified percutaneous endoscopic suturing to treat a fistula after a PEG removal [6]. Moreover, his group study used a combined approach of chemical and electric cautery and clip management and showed some promise in a case series [12]. Another recent study described an endoluminal method for the closure of a gastrogastric fistula that involves mechanical ablation of the mucosa of the fistula orifice with a mechanical closure of the stomach defect by using a novel permanent tissue apposition system (TAS) in four patients. All fistulas were closed, but a permanent closure could not be achieved [13]. 

In our paper, we described a novel method to close a gastrocutaneous fistula by the application of a new endoscopic clipping system (OTSC, Ovesco Endoscopy GmbH, Tuebingen, Germany). Currently, the indications for treatment with OTSCs are primary or postinterventional bleeding in the gastrointestinal tract, or closure of iatrogenic full-thickness or covered perforations [1]. The OTSC system can be used immediately when the lesion is seen. The correct and secure application depends on the correct fitting of the application cap to the lesion. Due to inadequate visual contact at the moment of application, the endoscopists must be sure that the position is correct. We chose the “traumatic” OTSC type with sharp teeth for achieving the best gripping of the fibrotic tissue around the gastric orifice. In previous reports, Repici et al. [14] and Kirschniak et al. [1] treated with the OTSC system 9 and 11 patients, respectively, with bleeding and/or deep wall lesions of the gastrointestinal tract. Moreover, Schurr et al. used the OTSC clip for closure of the gastrostomy after NOTES [15].

Traina et al. [16] recently reported that the OTSC device can be used to close a 10 mm tracheoesophageal fistula, while Conio et al. [17] have shown that a gastric fistula, occurring after sleeve gastrectomy, can be successfully closed using this technique. Furthermore, very recently, Iacopini et al. described the closure of two chronic gastrocutaneous fistulas with OTSC [18].

## 4. Conclusion

Our case adds to the previous literature demonstrating a role for OTSC clip in the closure of gastrocutaneous fistula. We believe that OTSC application is a safe and effective endoscopic method for the successful treatment of gastrocutaneous or esophageal fistulas. However, prospective comparative studies are needed to work out the sufficiency and efficacy of this new method regarding the gastrocutaneous fistulas closure, before this indication is added to the others existing for the OTSC application in interventional gastrointestinal endoscopy.
